# Huntingtin localisation studies - a technical review

**DOI:** 10.1371/currents.RRN1211

**Published:** 2011-02-16

**Authors:** Alis Hughes, Lesley Jones

**Affiliations:** School of Medicine, Cardiff University

## Abstract

It is well recognised that there are pitfalls when defining the subcellular localisation of a protein with immunocytochemistry. Accurate protein localisation to particular cellular micro-architecture is crucial in defining its role within the cell. Huntingtin (HTT), the protein mutated in the neurodegenerative disorder Huntington’s disease (HD) is a large protein of ill-defined function. Bearing little resemblance to other proteins, its function has been difficult to assign, therefore localising this protein with precision within the cell may provide further clues as to its normal and pathological function. Lack of consistency between methods employed in different studies has resulted in varying conclusions as to its subcellular localisation. This technical review investigates the effects that different immunocytological methods can have upon the apparent subcellular localisation of the huntingtin protein, and discusses the implications this may have.

## Introduction

Huntingtin (HTT) is the protein product of the *HD *gene which is the faulty gene in Huntington’s disease (HD).  The precise subcellular localisation of HTT has been the subject of much debate since the generation of the first anti-HTT antibodies following the cloning of the *HD* gene [1] [2] [3]. Establishing localisation is important for several reasons. Firstly, to refine HTT’s role within the cell; secondly, to track any protein or subcellular interactions with confidence and thirdly, to monitor any effects of post-translational modifications on the localisation of this protein. Endogenous HTT has been detected and localised in various cell lines and in human, mouse, rat, rabbit and monkey tissue, using various antibodies (Table 1).  

**Table 1: Anti-HTT antibodies used to detect and localise huntingtin**

**Table:** 

**Huntingtin antibody**	**Type**	**Region against which antisera raised**	**Reference**
Ab1356	Affinity purified rabbit pAb IgG	3114 – 3141 (aa)	1
Ab585	Rabbit pAb IgG	585 – 754 (aa)	8, 10, 16, 22, 23
Ab1	Affinity purified rabbit pAb	1 – 17 (aa)	8, 10, 12, 23, 24
Ab2911	Affinity purified rabbit pAb	2911 – 3140 (aa)	8
Ab2527	Affinity purified rabbit pAb	2527 – 2547 (aa)	8, 22
Ab1173	Rabbit pAb	1173 – 1196 (aa)	8
mAb4C8/mAb2166	Mouse mAb	181 - 810 (aa)	2, 7, 14, 25, 26
mAb4E6	Mouse mAb	4057 – 5253 (cDNA)	2
mAb2E8/mAb2168	Mouse mAb	2146 - 2541 (aa)	2, 7
mAb2C1	Mouse mAb	8366 – 9254 (cDNA)	2
AP78	Affinity purified rabbit pAb	1 – 17 (aa)	11, 27
AP81	Affinity purified rabbit pAb	650 – 663 (aa)	11
HDp549	Affinity purified rabbit pAb	549 – 679 (aa)	3, 14, 28
-	Rat mAb	549 – 679 (aa)	3
IT15 antibody	Affinity purified rabbit pAb	1188 – 1204 (aa)	4
GHM1	Mouse mAb	1765 – 2070 (aa)	26, 27, 28
BKP1	Affinity purified rabbit pAb	3 – 16 (aa)	25, 26, 27
BKP4	Affinity purified rabbit pAb	1593 – 1608 (aa)	26
mAb 2172	Mouse mAb	2683 – 2979 (aa)	26
AP194	Rabbit pAb	1 – 17 (aa)	7, 34
HDB4E10	Mouse mAb	1844 – 2131 (aa)	7, 17
HDC8A4	Mouse mAb	2703 – 2911 (aa)	7, 17
HDA3E10	Mouse mAb	997 – 1276 (aa)	17
Antibody 1356	Affinity purified rabbit pAb	3114 – 3141 (aa)	5, 6
Antibody 1354 / 1358	Affinity purified rabbit pAb	3114 – 3141 (aa)	5, 6
Antibody 1359	Affinity purified rabbit pAb	701 – 744 (aa)	5, 6
Antibody 1495	Affinity purified rabbit pAb	701 – 744 (aa)	5, 6
Antibody 93	Affinity purified rabbit pAb	1929 – 2421 (aa)	5, 6
Antibody 97	Rabbit pAb	596 – 1030 (aa)	6
Antibody 98	Rabbit pAb	852 – 1193 (aa)	6
Antibody 7	Affinity purified rabbit pAb	1 – 19 (aa)	6
Antibody 10	Affinity purified rabbit pAb	1 – 19 (aa)	6
Antibody 1859	Rabbit pAb	1 – 19 (aa)	6
Antibody 1862	Rabbit pAb	1 – 19 (aa)	6
Antibody 2	Rabbit pAb	11 – 19 (aa)	6
Antibody 5	Rabbit pAb	11 – 19 (aa)	6
Anti-HUNT3	Affinity purified rabbit pAb	2110 – 2121 (aa)	29
CAG53b	Rabbit pAb	1 – 118 (with 51Q) (aa)	24, 30
HF1	Rabbit pAb	1981 – 2580 (aa)	13, 14, 31, 32, 35
-	Rabbit pAb	3099 – 3130 (aa)	13
HP1	Rabbit pAb	80 – 113	31
HP12	Rabbit pAb	2 – 17	31
mAb 2170	Mouse mAb	1247 – 1645 (aa)	8
mAb 2168	Mouse mAb	2146 – 2541 (aa)	8
mAb 1574	Mouse mAb	Expanded polyQ (TBP*) (aa)	8, 34
Ab78	Rabbit pAb	1 – 17 (aa)	24
HD1	Rabbit pAb	1 – 17 (aa)	24
HD48	Rabbit pAb Mouse mAb	1 – 82 (aa)	32
EM48	Rabbit pAb	1 – 256 (aa)	28, 33, 35, 36
4h7h7	Mouse mAb	1-149(mouse) 1-171(human) [detects polyQ]	9
Ab675	Affinity purified Rabbit pAb	1 – 17 (aa)	21
P1874/3B5H10	Mouse mAb	1 – 171 with 65Q (aa)	37
mAb5492(2B4)	Mouse mAb	1 – 82 (aa)	38
MW1	Mouse mAb	DRPLA**-19Q (aa)	39
MW2	Mouse mAb	DRPLA-35Q (aa)	39
MW3	Mouse mAb	HD exon 1 with 67Q (aa)	39
MW4	Mouse mAb	HD exon 1 with 67Q (aa)	39
MW5	Mouse mAb	DRPLA-35Q (aa)	39
MW6	Mouse mAb	HD exon 1 with 67Q (aa)	39
MW7	Mouse mAb	HD exon 1 with 67Q (aa)	39
MW8	Mouse mAb	HD exon 1 with 67Q (aa)	39

Immunofluorescent studies with cells in culture have supported both a cytoplasmic and nuclear localisation of HTT [4] [5] [6] [7] [8].  The antibodies employed in these studies have spanned the entire length of the HTT protein (as shown in Table 1) thus indicating that the nuclear and cytoplasmic immunostaining seen does not appear to correspond to any particular HTT fragment, but rather the full length protein, or possibly a mixture of fragments such as those described in the recent study of Landles *et al*, 2010 [9].  Most of these reports have also employed the complementary technique of subcellular fractionation corroborating the presence of HTT within the cytoplasmic and nuclear fractions of the cells tested [5] [6] [7] [8].  Moreover, in a subgroup of cell types, an array of HTT cleavage products were detected in the nucleus, and in some instances in the cytoplasm, that appeared to be both cell type and species dependent [7] [8].  More recently, the nature of such fragments has been studied in further detail in the *Hdh*Q150 knock-in mouse brain [9]. It is possible that HTT fragments could be differentially located within the cell and indeed, could have individual functions.  

In tissue, HTT mainly presents as an exclusively cytoplasmic protein [2] [3] [10] [11] [12] [13] [14] [15], although a few immunohistochemical (IHC) reports also show HTT within nuclei [1] [8] [16] [17].  Factors that may influence the final immunostaining pattern include the antibody used, the fixation and permeabilisation employed, the specific tissues studied and the post-mortem intervals in IHC studies of human tissue. HTT may also adopt different conformations according to cell type or stage of cellular differentiation, thereby evading detection or presenting different epitopes under different conditions.   

It is therefore apparent that numerous variables can affect the immunostaining result obtained [18] [19].  Sample preparation requires fixation, to maintain cell structure and prevent antigen leakage, and permeabilisation of cells to allow antibodies access to the specific antigens [20].  This study focuses on the effect that the immunocytological methods of fixation and permeabilisation have on the apparent subcellular localisation of the HTT protein.  We examined both main types of fixation method.  Organic solvents such as alcohols and acetone remove lipids and dehydrate the cells thereby precipitating the proteins on the cellular architecture.  As this has a simultaneous permeabilisation action, the need for a further permeabilisation step is removed.  Cross-linking reagents such as paraformaldehyde and glutaraldehyde form intermolecular bridges between proteins and create a network of linked antigens.  These do not permeabilise the cell so a further permeabilisation step with either an organic solvent or a non-ionic detergent is used [20]. Here, five different methods of fixation/permeabilisation were compared to investigate the apparent localisation of the HTT protein in cells in culture.

## Materials and methods

**R6/1 mouse breeding and primary striatal neuron dissection**

 CBAxC57/BL6 female mice (Harlan Olac) were bred with R6/1 male mice (in-house colony). Pregnant female mice at gestational stage E13-15 were killed and the uterine horns removed and transferred to ice cold DMEM (Invitrogen).  All experiments were conducted in accordance with the United Kingdom Animals (Scientific Procedures) Act of 1986, and local ethical review. 

 For the dissection of primary neurons, tissue was removed from the whole ganglionic eminence and transferred to eppendorf tubes containing sterile Hanks Buffered Salt Solution (HBSS). Dissected tissue was enzymatically digested using a solution of 0.1% trypsin (Worthington) and 0.05% DNase (Sigma-Aldrich) at 37oC for 20 minutes. Following two rinses in DMEM/F12 containing 0.05% DNase the tissue was dissociated to a single cell suspension by mechanical trituration using a Gilson pipette. Cell counts and percentage viable were assessed by dilution of cell suspensions in trypan blue and counted under an Olympus stereo microscope using a standard haemocytometer grid. 

**Cell culture**

HeLa cells were grown on coverslips in 6 well plates in complete DMEM consisting of DMEM, penicillin/streptomycin (100unitsml^-1^/100μgml^-1^) (Invitrogen), glutamine (2mM) (Invitrogen) and 10% fetal bovine serum (FBS) (Invitrogen).  IMR32 cells were grown on poly-Lysine coated coverslips in 6 well plates in complete RPMI medium consisting of RPMI medium (Invitrogen), penicillin/streptomycin (100unitsml^-1^/100μgml^-1^), glutamine (2mM) and 5% FBS.  

To culture primary striatal cells from R6/1 offspring, 150,000 cells per 100μl were plated onto laminin-coated coverslips in 6 well plates.  Standard growth media consisted of neurobasal medium (Invitrogen) supplemented with B27 nutrient matrix (1%) (Invitrogen), antibiotic/antimycotic solution (1%) (Invitrogen), Glucose (30mM) (Sigma-Aldrich), glutamine (1mM) and 1% FBS. Cells were allowed to adhere and grow for 7 days prior to processing. 

**Immunostaining**

Five different fixation/permeabilisation methods were employed to assess the contribution of methodology to the immunostaining pattern of HTT observed.  These are summarised in Table 2. 

- **Table 2: Fixation and permeabilisation methods used to study the distribution of huntingtin with Ab 675**

**Table:** 

**Method**	**Fixation method**	**Permeabilisation method**
A	4% paraformaldehyde for 15 mins at room temperature	0.1% Triton-X-100 for 30 mins at room temperature
B	-10^o^C methanol for 5 mins: Air dry (rehydrate in Phosphate Buffered Saline (PBS) before processing)	-
C	Ice cold Acetone for 2 mins: Air dry (rehydrate in PBS before processing)	-
D	0.25% paraformaldehyde for 15mins followed by 70% ethanol for 1 hour	0.1% Triton-X-100 for 30 mins at room temperature
E	4% paraformaldehyde for 15 mins at room temperature	0.5% Triton-X-100 for 2 mins at room temperature

The primary antibody used to detect HTT was Ab 675, a rabbit polyclonal raised against the N-terminal region (N1-17) of HTT [21].  Following fixation and permeabilisation, cells were blocked in 10% goat serum at room temperature for 2 hours prior to incubation with rabbit polyclonal antibody 675 (1:100 dilution) for 1 hour at 37^o^C.  Goat anti-rabbit secondary antibody alexafluor 568 (1:100 dilution) was subsequently incubated with the cells for 1 hour at 37^o^C, prior to overnight storage in PBS at 4^o^C and processing for microscopy.  In order to control for false positive results, primary antibody was omitted and the secondary antibody only was added. 

**Fluorescence microscopy**

Cells processed for immunocytochemical analysis were visualised using a Zeiss Axiovert-S100 TV fluorescence microscope (Zeiss) using an excitation 560/40 / emission 630/60 filter.  Optimal magnification of the cell images was achieved using immersion oil and an apical zoom lens (x40). 

**Confocal laser scanning microscopy (clsm)**

A 1024-MP laser scanning microscope (lsm) (BioRad Microscience) was used to obtain optical sections through different cells immunostained with antibody 675.  The 1024 scan head was attached to a Zeiss Axiovert-S100 TV microscope. This instrument was used in two modes 1) EGFP confocal mode using a visible krypton-argon ion laser (AL) at 488nm and 2) ECFP-multiphoton mode using a pulsed infrared laser 5w Verdi-mira-900 (Coherent Ltd, UK) at 780nm.  A selection of typical high-resolution (x,y) single frames (512 x 512; 0.035μm x 0.035μm pixel size) were collected with a x40 1.3 NA oil immersion lens.  

## **Results**

HTT immunostaining in HeLa cells with various methods of cell processing 

**Figure:**
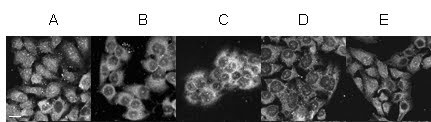

**Figure 1 : Immunolocalisation of endogenous huntingtin within HeLa cells using various methods of cell processing**

- Hela cells treated with different methods of fixation and permeabilisation (Table 2) displayed varying patterns of HTT localisation.  Nuclear localisation was more prominent when cross-linking fixation methods were used, and diminished when more dehydrating methods were employed.  Cells were incubated with Ab 675 (1:100) and a goat-anti-rabbit-568 secondary antibody (1:100) prior to visualisation by confocal microscopy (CLSM).   Scale bar = 10µm.

*Method A:*

HeLa cells fixed with 4% paraformaldehyde and permeabilised with 0.1% Triton-X-100, exhibited HTT immunostaining with Ab 675 in both the cytoplasm and nucleus in approximately 85-90% of cells studied. The remaining 10-15% of cells exhibited an exclusively cytoplasmic stain (Figure 1A).  The overall appearance was very granular with numerous bright punctae interspersed throughout the whole cell.  The discrete punctae were more pronounced when studied by clsm due to the higher resolution.  In the majority of cells, immunostaining was of equal intensity in both cytoplasmic and nuclear compartments, and within the nucleus, there was an intense labelling of punctate bodies.        

*Method B*

HeLa cells fixed and permeabilised with 100% ice-cold methanol demonstrated a predominantly cytoplasmic HTT localisation and only faint staining of the nucleoplasm (Figure 1B).  Brightly stained punctate bodies were noted within the nucleus.  Dehydration of the cells was apparent given their shrunken appearance.  

*Method C*

Treatment with ice-cold acetone also resulted in an apparent absence of HTT from the nucleoplasm and the localisation of HTT to punctate bodies within the nucleus.  Cytoplasmic staining of HTT was intense and more diffuse compared with the nucleus (Figure 1C).  However, cell morphology was adversely affected with enlarged nuclei and shrunken cytoplasm noted.    

*Method D*

A combination of both detergent and solvent was used in an attempt to preserve cellular architecture.  Cells were fixed in 0.25% paraformaldehyde for 15 mins at room temperature prior to a further fixation/permeabilisation in 70% ethanol for 1 hour and a subsequent permeabilisation step with 0.1% Triton-X-100.  Cell morphology appeared less distorted than for methods B and C.  There was an absence of diffuse HTT staining from the nucleoplasm yet it appeared lightly speckled and HTT localised to punctate nuclear bodies.  The immunostain within the cytoplasm was granular and punctate (Figure 1D).      

*Method E*

Cells fixed in 4% paraformaldehyde and permeabilised more vigorously with 0.5% Triton-X-100 for 2 minutes acquired a peculiar appearance.  Some cells maintained a diffuse nuclear immunostain whereas other cells had nuclei completely devoid of HTT signal.  HTT was seen to collapse into a thin perinuclear ring (Figure 1E).      

It is noteworthy that in each case, immunostaining with secondary antibody alone produced no significant stain thereby excluding a contribution from the secondary antibody in the staining pattern produced (data not shown).

**HTT immunostaining in IMR32 cells and mouse primary striatal neurons**

To extend this investigation, the immunostaining of HTT within IMR32 cells that had been fixed and permeabilised by methods A and C respectively was performed. This compared a cross-linking method with a dehydration method.  

This supported the finding of a fixation method-dependent effect upon the immunostaining pattern observed.  Acetone fixation created much background debris and little nuclear staining (Figure 2B), whereas paraformaldehyde resulted in a nuclear and punctate cytoplasmic stain (Figure 2A).

**Figure:**
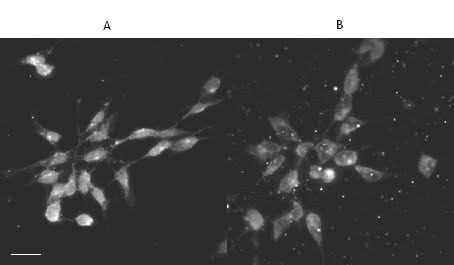

 **Figure 2 : Immunolocalisation of endogenous huntingtin within IMR32 cells fixed by two different methods**

IMR32 cells were fixed in 4% paraformaldehyde and permeabilised in 0.1% Triton-X-100 for 30 mins (A) or fixed in ice cold acetone for 2 mins (B).  Acetone fixation created much background debris and little nuclear staining (B), whereas paraformaldehyde fixation showed apparent localisation of HTT within the nucleus  and a punctate pattern in the cytoplasm (A).  Cells were incubated with Ab 675 (1:100) and a goat-anti-rabbit-568 secondary antibody (1:100) prior to visualisation on clsm.  Scale bar = 20mm.   

 Similarly, in mouse primary striatal neurons, HTT immunostaining varied with the immunocytochemical method employed.  The fixation protocol was critical for these cells to maintain their structure and adherence to the coverslip.  Cells were fixed in 4% paraformaldehyde for 15 mins at room temperature in all cases, however, the subsequent permeabilisation method was varied.  Cells were permeabilised with either Triton-X-100 (0.1%) for 30 mins at room temperature or 100% ethanol for 2 mins (Figure 3).

**Figure:**
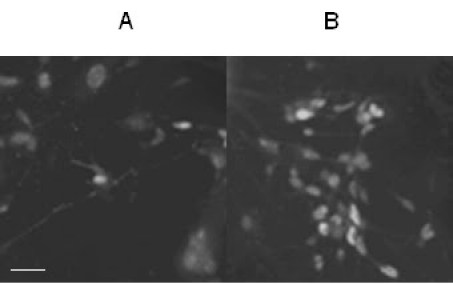

**Figure 3 : Immunolocalisation of endogenous huntingtin within primary striatal neurons permeabilised by two different methods**

Mouse primary striatal neurons were fixed in 4% paraformaldehyde and permeabilised with either 0.1% Triton-X-100 (A) or 100% ethanol (B).  The HTT immunostaining pattern observed was similar for both permeabilisation reagents, as it appeared diffusely in both the cytoplasm and nucleus (A, B).  However, it exhibited a more punctate pattern in axons when treated with Triton-X-100 (A).  Cells were incubated with antibody 675 (1:100) in conjunction with the goat-anti-rabbit-568 secondary antibody (1:100) and visualised by clsm.    Scale bar = 20mm.  

The immunostaining pattern observed was similar when compared between different permeabilisation methods with HTT appearing in both the cytoplasm and nucleus, and the nuclear signal being more intense than that of the cytoplasm (Figure 3A, B).  However, permeabilisation with Triton-X-100 led to a more punctate pattern within axons (Figure 3A) compared with the ethanol treatment (Figure 3B).  This demonstrated that the choice of permeabilisation reagent can also have an effect on the apparent localisation of HTT observed, in addition to the fixative used. 

## Discussion

Details of the precise subcellular localisation of the HTT protein remain unclear and the varying methodologies employed to address this issue create more uncertainty. Most studies support the finding that HTT exists in both the cytoplasm and nucleus, although discrepancies do exist and the exact nature of the protein in each compartment remains ill defined.  It is well established that the quality of the immunostaining pattern obtained is dependent upon numerous factors [19] and this brief study investigates the effect of fixation and permeabilisation methods on the resultant subcellular localisation of HTT.  A significant effect was demonstrated whereby the nuclear localisation was more prominent when cross-linking fixation methods were used, and diminished when more dehydrating methods were employed.  This was recognised for both HeLa and IMR32 cells and is consistent with previous immunostaining studies with HTT in purkinje cells of rabbit cerebellum [17] and in primary human fibroblasts [8]. Although the purpose of this study was not to compare HTT localisation between cell types, it is noteworthy that the pattern of distribution of HTT differed between cell types.  A similar conclusion was drawn in the study of De Rooij *et al.*, 1996 [5].

 We have also shown that certain methods can lead to obvious cellular damage and therefore questionable validity of some of the patterns observed.  The method of choice would therefore be a compromise between preservation of the cellular microarchitecture whilst retaining the epitopes of interest [18] [20].  

This study confirms that immunostaining patterns should be interpreted with caution and related closely to the methodology employed where a lack of consistency is found between studies.  Using additional methods for corroboration such as live cell overexpression studies and subcellular fractionation, may help support findings though these too have their own issues of validity.  Accurate subcellular localisation of HTT is important to define its role within the cell both normally and pathologically and so an awareness of the effects of varying fixation and permeabilisation methods on this localisation is important.  Methodology may disturb protein conformation and function and this could lead to false conclusions being drawn from artifactual findings.     

## Acknowledgements

We thank Lyn Elliston for technical assistance, Rachel Errington for assistance with confocal laser scanning microscopy and Rosemary Fricker-Gates for her help with primary striatal neuron preparation.    

## Funding information

This study was supported by a MRC studentship to ACH.

## Competing interests

The authors have declared that no competing interests exist.   
